# mTOR-S6K1 pathway mediates cytoophidium assembly

**DOI:** 10.1016/j.jgg.2018.11.006

**Published:** 2019-02

**Authors:** Zhe Sun, Ji-Long Liu

**Affiliations:** a School of Life Science and Technology, ShanghaiTech University, Shanghai, 201210, China; b MRC Functional Genomics Unit, Department of Physiology, Anatomy and Genetics, University of Oxford, Oxford, OX1 3PT, United Kingdom

**Keywords:** mTOR, Cytoophidium, CTP synthase, Colorectal cancer cell, *Drosophila*, ATF4, activating transcription factor 4, CA-S6K1, constitutively active S6K1, CTPS, cytidine triphosphate synthase, DON, 6-Diazo-5-oxo-L-norleucine, EL, everolimus, IMPDH, inosine monophosphate dehydrogenase, MTHFD2, methylenetetrahydrofolate dehydrogenase 2, mTOR, mechanistic target of rapamycin, mTORC1, mTOR complex 1, mTORC2, mTOR complex 2, Rap, rapamycin, Raptor, regulatory associated protein of mTOR complex 1, Rictor, RPTOR independent companion of mTOR complex 2, S6K1, ribosomal protein S6 kinase beta-1, UAS, upstream activation sequence

## Abstract

CTP synthase (CTPS), the rate-limiting enzyme in *de novo* CTP biosynthesis, has been demonstrated to assemble into evolutionarily conserved filamentous structures, termed cytoophidia, in *Drosophila*, bacteria, yeast and mammalian cells. However, the regulation and function of the cytoophidium remain elusive. Here, we provide evidence that the mechanistic target of rapamycin (mTOR) pathway controls cytoophidium assembly in mammalian and *Drosophila* cells. In mammalian cells, we find that inhibition of mTOR pathway attenuates cytoophidium formation. Moreover, CTPS cytoophidium assembly appears to be dependent on the mTOR complex 1 (mTORC1) mainly. In addition, knockdown of the mTORC1 downstream target S6K1 can inhibit cytoophidium formation, while overexpression of the constitutively active S6K1 reverses mTOR knockdown-induced cytoophidium disassembly. Finally, reducing mTOR protein expression results in a decrease of the length of cytoophidium in *Drosophila* follicle cells. Therefore, our study connects CTPS cytoophidium formation with the mTOR signaling pathway.

## 1 Introduction

CTP not only serves as the building blocks for nucleic acid synthesis, but also contributes to the synthesis of membrane phospholipids and protein sialylation (Huang and Graves, 2003; Higgins et al., 2007). Low intracellular concentration makes CTP one of the rate-limiting molecules for nucleic acid biosynthesis and other CTP-dependent events (Traut, 1994). Therefore, understanding the precise control of CTP production is crucial for cell metabolism and many growth-related processes.

CTP can be generated through either the *de novo* synthesis pathway or the salvage pathway in mammalian cells. CTP synthase (CTPS) is the rate-limiting enzyme that catalyzes the conversion of UTP to CTP using glutamate or ammonia as the nitrogen source (Levitzki and Koshland, 1971). It has been demonstrated in a number of studies that CTPS can be assembled into filamentous structures, termed cytoophidia, in several different organisms, including fruit fly, bacteria, yeast and mammalian cells (Ingerson-Mahar et al., 2010; Liu, 2010; Noree et al., 2010; Carcamo et al., 2011; Chen et al., 2011).

Recent studies have established a link between cytoophidium and CTPS enzymatic activity (Aughey et al., 2014; Barry et al., 2014; Noree et al., 2014; Strochlic et al., 2014; Lynch et al., 2017). In *Drosophila,* inhibition of the proto-oncogene *Cbl* disrupts cytoophidium formation, and the protein level of the oncogene *c-Myc* is correlated with cytoophidium abundance and size (Wang et al., 2015; Aughey et al., 2016). Moreover, CTPS activity was found to be elevated in various cancers such as hepatoma and lymphoma (Williams et al., 1978; Ellims et al., 1983). Recently, we also observed the presence of CTPS cytoophidia in a variety of human cancer tissues (Chang et al., 2017). These findings suggest that the formation of cytoophidia is an evolutionarily conserved property of CTPS.

In mammals, the mechanistic target of rapamycin (mTOR) is the key serine/threonine protein kinase, which can interact with several proteins to form two distinct molecular complexes, called mTOR complex 1 (mTORC1) and mTOR complex 2 (mTORC2) (Saxton and Sabatini, 2017). mTORC1 controls cell growth and metabolism by regulating protein synthesis, lipid and glucose metabolism, and protein turnover (Saxton and Sabatini, 2017). In contrast, mTORC2 regulates cell proliferation and survival primarily through phosphorylating Akt and several members of the AGC (PKA/PKG/PKC) family of proteins (Sarbassov et al., 2005; Saxton and Sabatini, 2017). Deregulation of the mTOR signaling pathway is associated with a number of human diseases, including cancer, type 2 diabetes, obesity, and neurodegeneration (Saxton and Sabatini, 2017).

Recent studies have established a direct link between mTOR pathway and nucleotide metabolism (Ben-Sahra et al., 2013, 2016; Robitaille et al., 2013). In this study, to get a better understanding of the regulation of cytoophidium, we used a human cancer cell line and *Drosophila* as model systems to investigate the regulation of cytoophidium assembly by mTOR. We show that inhibiting mTOR pathway results in cytoophidium disassembly without affecting CTPS protein expression. In addition, the mTOR pathway controls CTPS cytoophidium assembly mainly via the mTORC1/S6K1 signal axis. Thus, this study links mTOR-S6K1 pathway to the polymerization of the pyrimidine metabolic enzyme CTPS.

## 2 Results

### 2.1 mTOR regulates CTPS cytoophidium assembly

**Fig. 1:**
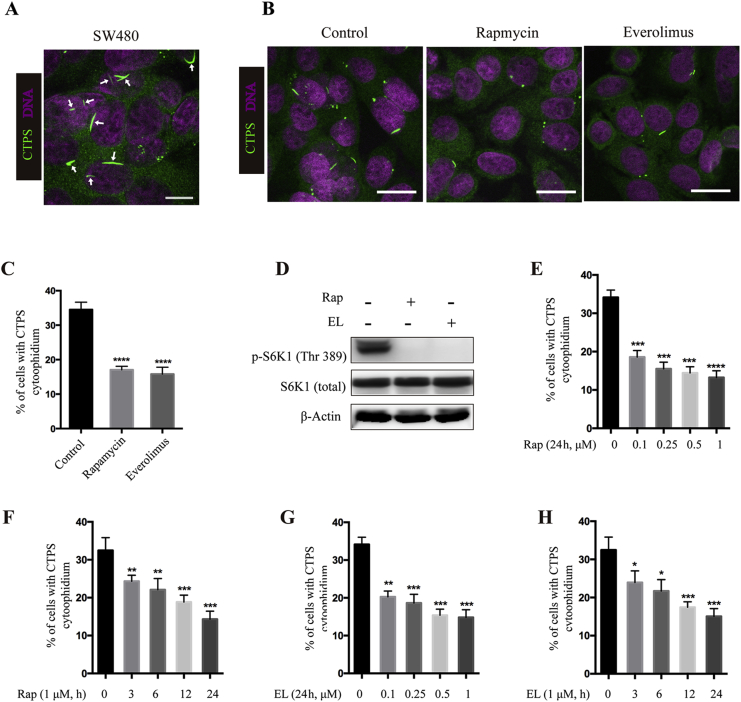
mTOR Inhibitors reduce cytoophidium formation. **A**: CTPS forms cytoophidium in SW480 cell. SW480 cells were cultured under normal culture conditions for 48 h and then fixed and subjected to immunofluorescence analysis with anti-CTPS antibody (green, arrow). Scale bar: 10 μm. **B‒D**: Pharmacologic inhibition of mTOR pathway reduces cytoophidium assembly. **B**: SW480 cells treated with vehicle (control) or 1 μM rapamycin or everolimus for 24 h were stained with anti-CTPS antibody. Scale bars = 20 μm. **C**: Percentages of SW480 cells with CTPS cytoophidia shown in (**B**). **D**: Immunoblotting analysis of the expression of p-S6K1 and total S6K1 upon rapamycin or everolimus treatment. **E‒H**: Dose and time effects of rapamycin and everolimus on cytoophidium formation. SW480 cells treated with the indicated concentration and time of rapamycin (**E** and **F**) or everolimus (**G** and **H**) were stained with anti-CTPS antibody. Percentages of SW480 cells with CTPS cytoophidium were quantified. Mean ± S.E.M., *P < 0.05; **P < 0.01; ***P < 0.001; ****P < 0.0001 *versus* control. One of four to seven similar experiments is shown. Rap, rapamycin; EL, everolimus.

**Fig. S1:**
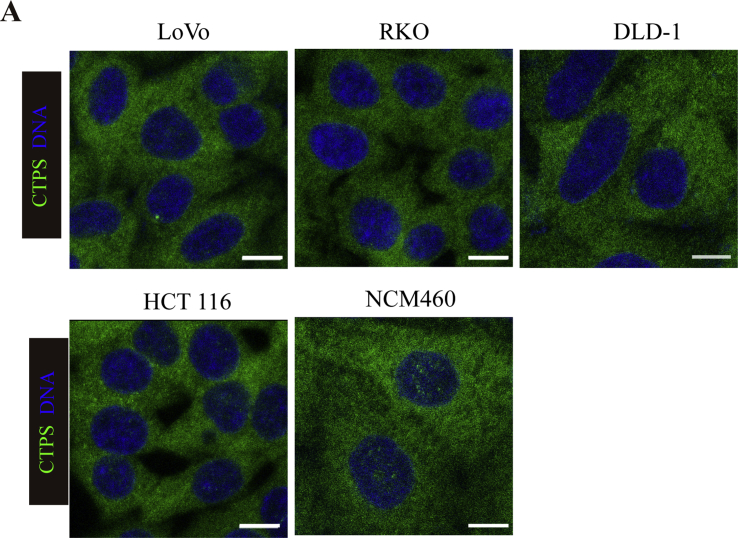
**The distribution of CTPS in other colorectal cancer and colon mucosal epithelial cells.** The indicated cell lines were cultured under normal culture conditions for 48 h and then fixed and subjected to immunofluorescence analysis with anti-CTPS antibody (green). Nuclei were labeled with DAPI (blue). Scale bar: 10 μm. DAPI, 40,6-diamidino-2-phenylindole.

To investigate whether the mTOR pathway regulates CTPS cytoophidium formation, we screened various cell lines. We observed that CTPS cytoophidia were present in ∼40% SW480 (a human colorectal cancer cell line) cells under normal culture conditions (Fig. 1A). However, it is hard to detect cytoophidia in other colorectal cancer cell lines, including LoVo, RKO, DLD1, HCT116 and a normal human colon mucosal epithelial cell line NCM460 (Fig. S1). Therefore, we used the SW480 cell line as a model for investigating the correlation between the CTPS cytoophidium and mTOR pathway activity.

**Fig. S2:**
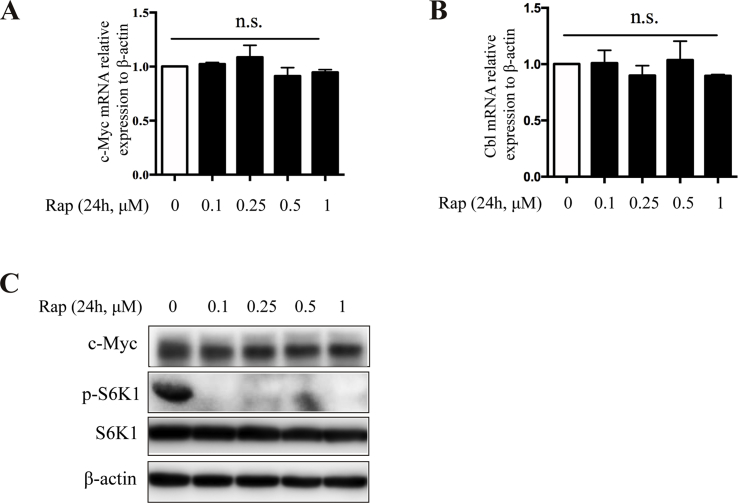
**The impacts of rapamycin treatment on c-Myc and Cbl expression. A and B:** SW480 cells treated with the indicated concentration of rapamycin for 24 h were subject to RT-PCR analysis for the mRNA expression of *c-Myc* (**A**) and *Cbl* (**B**), or were analyzed by immunoblotting for the protein expression of c-Myc (**C**).

We first treated SW480 cells with the mTOR inhibitors rapamycin or everolimus, and then labeled CTPS with anti-CTPS antibody. Immunofluorescence analysis showed that CTPS cytoophidia were present in 34.6% of control cells, while the percentage of cells with CTPS cytoophidia was reduced to 17% and 15.8% upon rapamycin or everolimus treatment, respectively (Fig. 1B and C). Inhibition of mTOR pathway was confirmed by the decreased level of phosphorylation at T389 of S6K1, a marker of active mTOR signaling (Fig. 1D). Further analysis showed that rapamycin and everolimus inhibit CTPS cytoophidium formation in a time- and dose-dependent manner (Fig. 1E‒H). Previous studies have shown that Myc and Cbl regulate cytoophidia formation in *Drosophila.* Here we investigate if mTOR mediates cytoophidium assembly through the reduction of c-Myc or Cbl. Our data showed that the mRNA levels of *c-Myc* and *Cbl* were not changed when cells were treated with rapamycin (Fig. S2A and B). Moreover, the protein levels of c-Myc were not changed upon rapamycin treatment either (Fig. S2C), suggesting that mTOR does not regulate cytoophidium formation via c-Myc or Cbl.

**Fig. 2:**
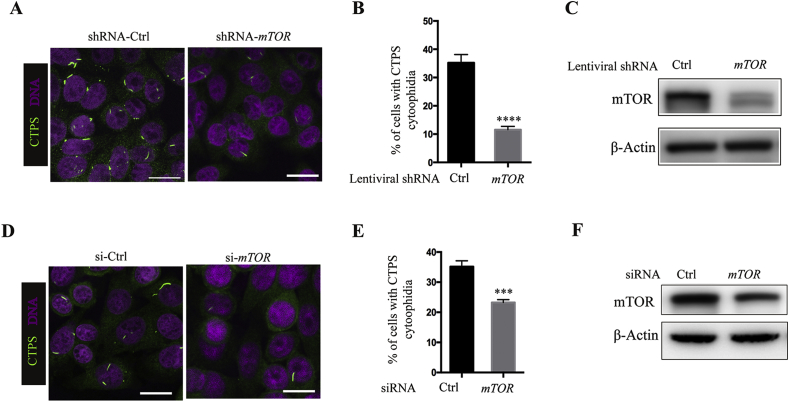
Knockdown of mTOR attenuates cytoophidium assembly. **A:** SW480 cells stably expressing shRNA targeting *mTOR* or *RFP* (as control) were subjected to immunofluorescence analysis with anti-CTPS antibody. **B:** Statistical analysis of the percentage of cells bearing CTPS cytoophidia in **(A)**. **C:** Western analysis of mTOR in cells expressing control or *mTOR* shRNA. **D:** SW480 cells were transfected with *mTOR* siRNA or scrambled control siRNA for 48 h and cells were subjected to immunofluorescence analysis with anti-CTPS antibody. **E:** Percentages of SW480 cells with CTPS cytoophidia shown in **(D)**. **F:** Western blotting analysis of mTOR protein expression in cells transfected with scrambled control siRNA or *mTOR* siRNA. Scale bars = 20 μm. β-Actin was used as a loading control. Mean ± S.E.M., ****P < 0.0001 *versus* control. One of four to seven similar experiments is shown.

**Fig. 3:**
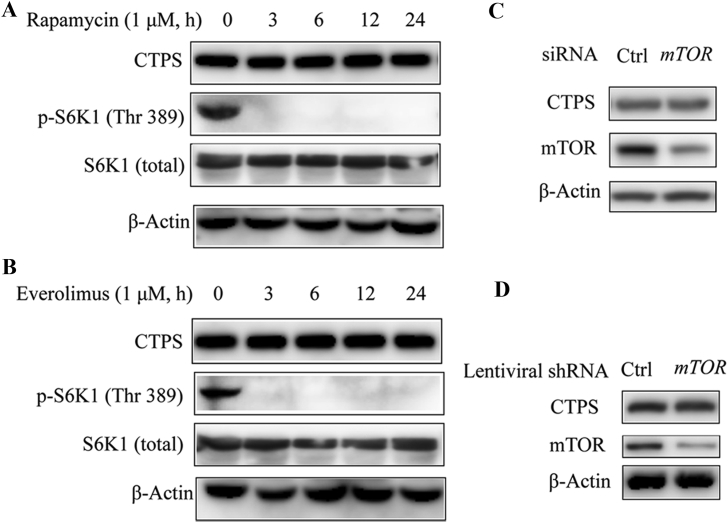
mTOR pathway inhibition does not alter CTPS protein levels. **A** and **B**: Effect of rapamycin **(A)** and everolimus **(B)** on CTPS protein expression. SW480 cells were treated with 1 μM rapamycin or everolimus for the indicated time. Cell lysates were analyzed by immunoblotting using anti-CTPS, anti-p-S6K1, and total S6K1 antibody. **C:** SW480 cells transfected with siRNA targeting *mTOR* or scrambled control siRNA were subjected to immunoblotting analysis with appropriate antibodies. **D:** SW480 cells stably expressing shRNA targeting *mTOR* or *RFP* (as control) were subjected to immunoblotting analysis with appropriate antibodies. β-Actin was used as a loading control. Data shown are representative of three independent experiments.

To confirm the correlation between mTOR pathway and cytoophidium assembly, we constructed a stable cell line expressing shRNA targeting *mTOR* and investigated the impact of *mTOR* knockdown on cytoophidium formation. Immunofluorescence results showed that the percentage of cells with CTPS cytoophidia dramatically decreased in cells expressing *mTOR* shRNA in comparison with the cells expressing control shRNA (Fig. 2A and B, 35.2% *versus* 11.5%; *P* < 0.0001). *mTOR* knockdown efficiency was confirmed by the decreased protein level of mTOR (Fig. 2C). A similar result was observed in an *mTOR* siRNA experiment. Compared with control siRNA, transfection of *mTOR* siRNA decreased the expression of mTOR protein (Fig. 2F), which was accompanied by a reduced proportion of cells presenting the CTPS cytoophidia (Fig. 2D and E, 35.4% *versus* 23.2%; *P* < 0.0001). The expression level of CTPS has been recognized as a critical factor for cytoophidium assembly (Ingerson-Mahar et al., 2010; Chen et al., 2011; Azzam and Liu, 2013; Liu, 2016). We next determined whether mTOR pathway inhibition reduces cytoophidium assembly through decreasing CTPS protein expression. Our data showed that neither rapamycin nor everolimus treatment affected CTPS protein expression (Fig. 3A and B). Inhibition of mTOR pathway was confirmed by the decreased level of phosphorylation at T389 of S6K1. In addition, knockdown of *mTOR* either by siRNA or by shRNA did not decrease CTPS protein expression (Fig. 3C and D).

### 2.2 mTORC1 controls CTPS cytoophidium assembly

**Fig. 4:**
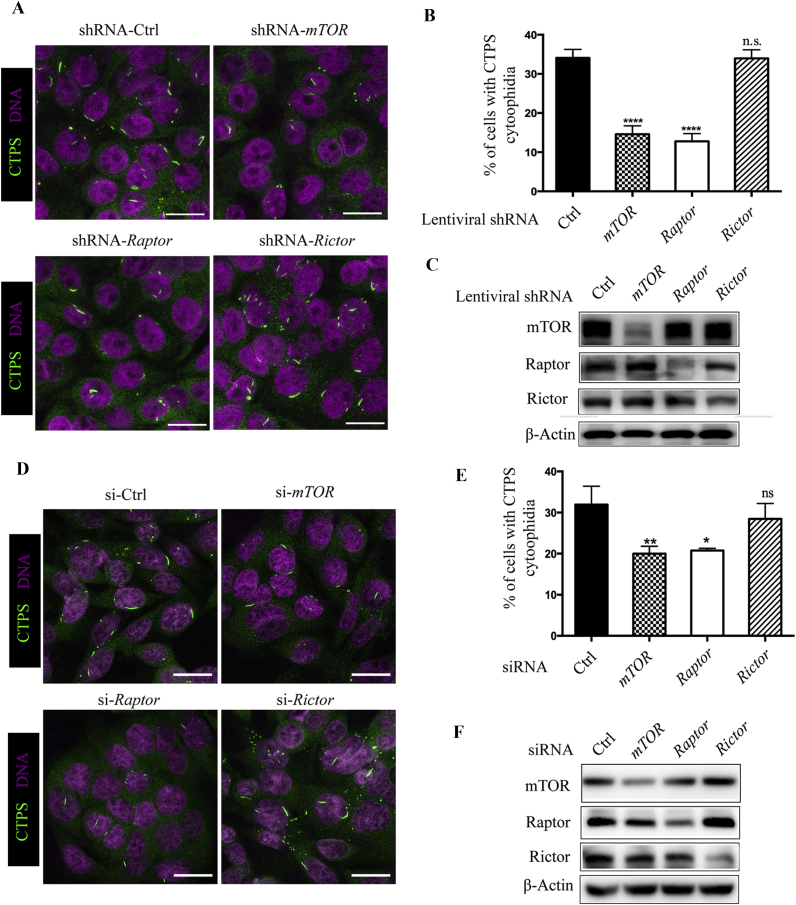
Reduced cytoophidium formation is dependent on mTORC1. **A**: SW480 cells expressing shRNA targeting *mTOR*, *Raptor*, *Rictor*, or *RFP* (as control) were analyzed by immunofluorescence staining for the presence of CTPS cytoophidium. **B**: Quantitative data of percentage of cells with cytoophidia shown in (**A**). **C**: Western blotting analysis of mTOR, Raptor and Rictor protein expression in the cells expressing shRNA of the indicated genes. **D**: SW480 cells transfected with siRNA targeting *mTOR*, *Raptor*, *Rictor*, or scrambled control siRNA were subjected to immunofluorescence staining with anti-CTPS antibody. **E**: Quantitative data for SW480 cells with CTPS cytoophidia shown in **(D)**. **F**: Western blotting analysis of mTOR, Raptor and Rictor protein expression in cells transfected with scrambled control siRNA or siRNA targeting *mTOR*, *Raptor* or *Rictor* with appropriate antibodies. β-Actin was used as a loading control in (**C**) and (**F**). Mean ± S.E.M., n.s., not significant; *P < 0.05, **P < 0.01; ****P < 0.0001 *versus* control. Scale bars = 20 μm. One of four to six similar experiments is shown.

mTOR can be incorporated into both mTORC1 and mTORC2, and is essential for them to exert their biological functions (Saxton and Sabatini, 2017). Rapamycin binds to FK506-binding protein 12 (FKBP12) and inhibits mTORC1 activity directly. Although the rapamycin-FKBP12 complex does not directly bind to and inhibit mTORC2, long-time rapamycin treatment attenuates mTORC2 signaling, likely because the rapamycin-bound mTOR cannot be incorporated into a new mTORC2 complex (Sarbassov et al., 2006). Therefore, we next determined which complex plays a dominant role in controlling CTPS cytoophidium assembly. For this purpose, we constructed two other stable cell lines expressing shRNA targeting a specific component of mTORC1 (Raptor) or a specific component of mTORC2 (Rictor) (Saxton and Sabatini, 2017). Immunofluorescence data showed that knockdown of *Rictor* did not change the proportion of cells with cytoophidia (Fig. 4A and B). In contrast, the percentage of cells presenting cytoophidia was reduced from 34.1% to 12.7% in *Raptor* knockdown cells as compared with control cells, and the degree of reduction is comparable to cells expressing *mTOR* shRNA (Fig. 4A and B). The knockdown efficiency was confirmed by Western blotting assay (Fig. 4C). For further confirmation of this phenomenon, we conducted a siRNA experiment. We found no difference in the percentage of cells with cytoophidia when cells were transfected with *Rictor* siRNA as compared with control siRNA. However, the transfection of *Raptor* siRNA significantly decreased the proportion of CTPS cytoophidia-positive cells from 32% to 20% (Fig. 4D and E), which is similar to the transfection of *mTOR* siRNA. The knockdown efficiency of the indicated genes was verified by Western blotting (Fig. 4F). Taken together, these results show that mTORC1 plays a dominant role in controlling CTPS cytoophidium assembly.

### 2.3 mTORC1 controls CTPS cytoophidium formation through S6K1

**Fig. 5:**
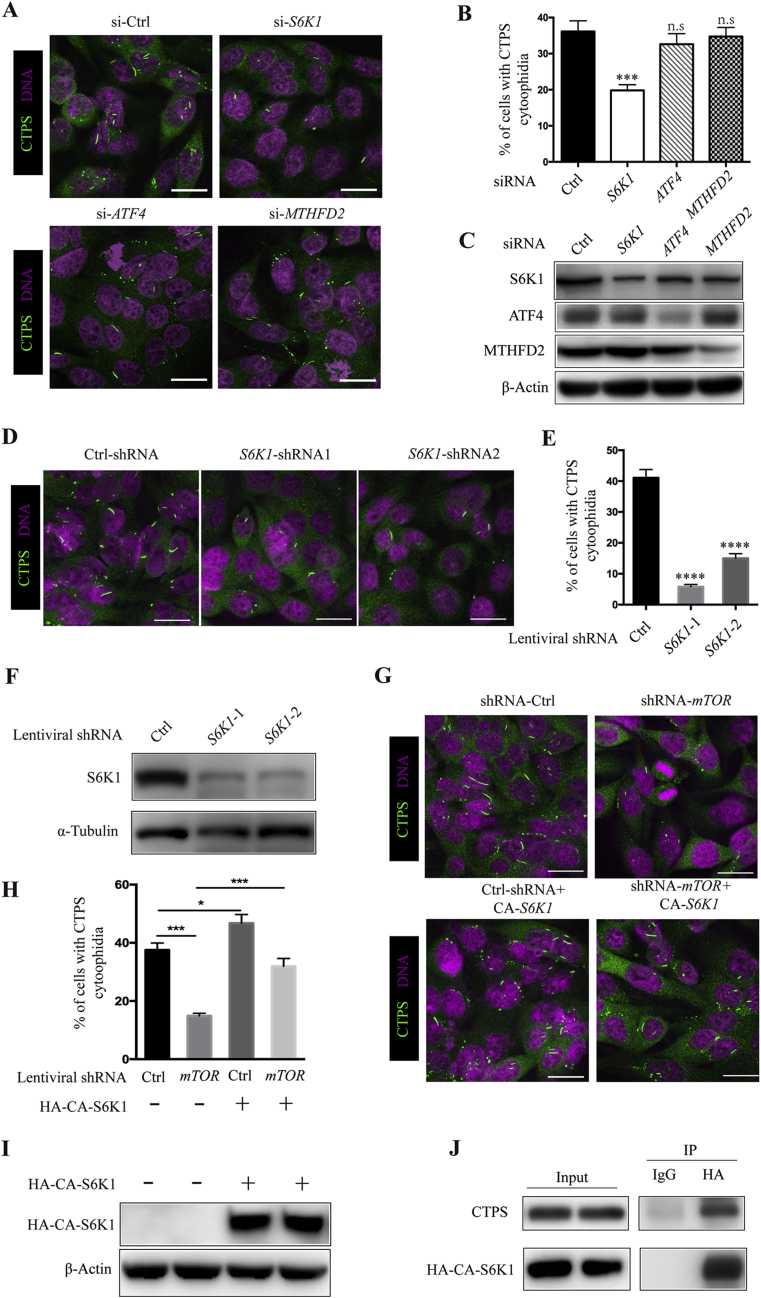
mTORC1 controls CTPS cytoophidium formation through S6K1. **A**: SW480 cells were transfected with siRNA targeting *S6K1*, *ATF4* or *MTHFD2*, or scrambled control siRNA for 48 h, and cells were subjected to immunofluorescence staining with anti-CTPS antibody. **B**: Percentages of SW480 cells with CTPS cytoophidia shown in (**A**). **C**: Western blotting analysis of S6K1, ATF4 and MTHFD2 protein expression in cells transfected with siRNA targeting the indicated genes. **D**: SW480 cells infected with two distinct shRNA targeting *S6K1* or *RFP* (as control) were analyzed by immunofluorescence staining for the presence of CTPS cytoophidium. **E**: Quantitative data of percentage of cells with CTPS cytoophidia shown in (**D**). **F**: Immunoblotting analysis of S6K1 expression in cells stably expressing shRNA targeting *S6K1* or *RFP.***G‒I**: Overexpression of CA-S6K1 reverses mTOR knockdown-induced reduction of cytoophidium formation. SW480 cells expressing control or *mTOR* shRNA were infected with lentiviruses expressing HA-CA-S6K1. The resulted cells were subjected to immunofluorescence (G and H) and Western blotting (**I**) analysis with appropriate antibodies and the percentages of cells bearing cytoophidium were quantified (**H**). **J**: SW480 cells stably expressing HA-CA-S6K1 were cultured for 48 h and the lysates prepared were subjected to immunoprecipitation by anti-HA antibody or normal mouse IgG and the presence of CTPS was examined by immunoblotting using anti-CTPS antibody. β-Actin and α-Tubulin were used as a loading control in (**C** and **I**) and (**F**), respectively. Mean ± S.E.M., n.s., not significant; **P < 0.01; ***P < 0.001; ****P < 0.0001 *versus* control. Scale bars = 20 μm. One of four to six similar experiments is shown.

Recent studies showed that mTORC1 could promote purine and pyrimidine synthesis through the ATF4/MTHFD2 and S6K1 pathway, respectively (Ben-Sahra et al., 2013, 2016). To further understand the mechanisms by which mTORC1 regulates CTPS cytoophidium formation, we analyzed the effects of *ATF4*, *MTHFD2* or *S6K1* knockdown on CTPS cytoophidium formation. In comparison with cells transfected with control siRNA, no significant difference in the percentage of cells with cytoophidia was observed in cells transfected with *ATF4* or *MTHFD2* siRNA. Yet, transfection of *S6K1* siRNA dramatically decreased the proportion of cells containing cytoophidia from 36.1% to 19.8% (Fig. 5A and B). Western blotting was used to verify the knockdown efficiency of the indicated genes (Fig. 5C). The role of S6K1 in cytoophidium assembly was further confirmed by lentiviral shRNA targeting *S6K1*. Immunofluorescence results showed that the percentage of cells expressing *S6K1* shRNA-1 or shRNA-2 which contained CTPS cytoophidia dropped significantly from 41.1% to 6% and 15%, respectively, in comparison with cells expressing control shRNA (Fig. 5D and E). Cells stably expressing *S6K1* shRNA-1 or shRNA-2 showed significantly reduced S6K1 protein expression (Fig. 5F). Next, we sought to examine whether overexpression of a constitutively active S6K1 (CA-S6K1) (Julien et al., 2010; Treins et al., 2010) could reverse the inhibitory effect of mTOR knockdown on CTPS cytoophidium assembly. Therefore, we stably overexpressed HA-CA-S6K1 in the cells expressing *mTOR* shRNA, and then analyzed cytoophidium assembly by immunofluorescence staining. As expected, knockdown of *mTOR* reduced the percentage of cells containing cytoophidia from 38% to 15%, while it rose to 32% in the cells stably expressing CA-S6K1 (Fig. 5G and H). Meanwhile, the expression of HA-CA-S6K1 alone increased the frequency of cells with cytoophidia from 38% to 46% (Fig. 5G and H). The expression of HA-CA-S6K1 was verified by Western blotting assay (Fig. 5I). We next determined if S6K1 could interact with CTPS. Our co-immunoprecipitation (Co-IP) data showed a clear interaction between HA-CA-S6K1 and CTPS (Fig. 5J). Thus, these data suggest that the mTOR pathway controls CTPS cytoophidium assembly mainly through S6K1 kinase and S6K1 may directly phosphorylate CTPS and regulate its filamentation.

### 2.4 mTOR is required for cytoophidium assembly in *Drosophila*

**Fig. 6:**
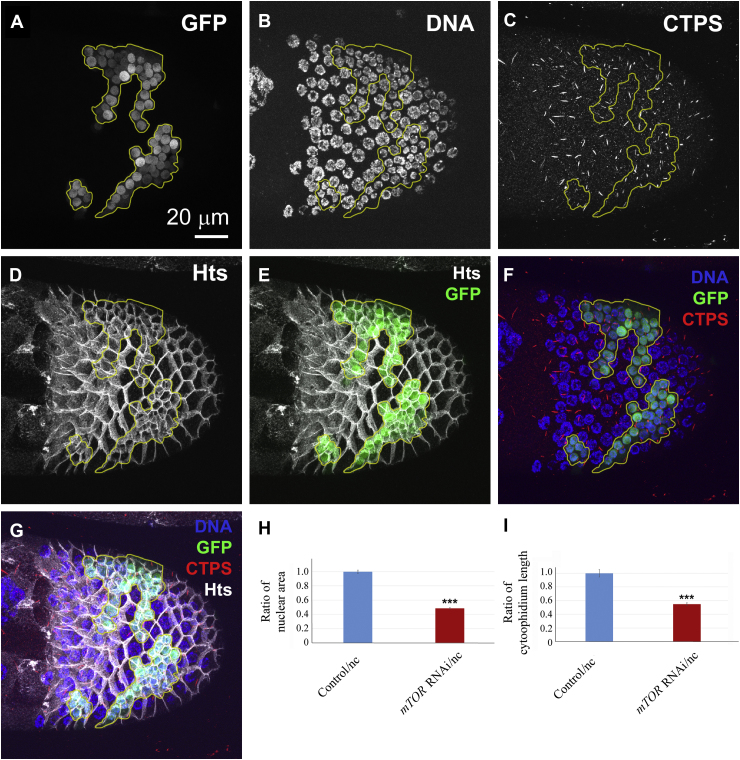
*mTOR* knockdown inhibits cytoophidium assembly in *Drosophila* follicle cells. **A** and **B**: UAS-mTOR-RNAi clones (i.e., *mTOR* RNAi) marked with GFP (**A**, outlined in yellow in **A‒G**) have decreased nuclear size **(B)**. **C, F** and **G**: *mTOR* knockdown cells have reduced length of cytoophidium as indicated by an antibody against CTPS. The cell boundary is outlined by membrane protein Hu-li tai shao (Hts) (**D**, **E** and **G**). **H**: Statistical analysis of the nuclear size in (**B**). Nuclear sizes are expressed as a ratio of the average nuclear size in GFP positive cells to neighbouring cells. **I:** Statistical analysis of the cytoophidium length of *mTOR* knockdown (*mTOR* RNAi) cells and their neighbouring control cells. Cytoophidium lengths are expressed as a ratio of the average cytoophidium length in GFP positive cells to neighbouring GFP negative cells. Mean ± S.E.M., ***P < 0.001. Over 60 cells were quantified from each group.

We further investigated the correlation between mTOR pathway and CTPS cytoophidium assembly in *vivo*. Two independent UAS driven shRNA were used to knock down the expression of mTOR in follicle cell epithelium of the *Drosophila* egg chambers. Compared with the neighboring cells, the cells expressing *mTOR* shRNA showed reduced nuclear size (Fig. 6A and B). The nuclear size in *mTOR* knockdown cells is less than 50% of that in neighboring cells (Fig. 6H), which is in agreement with the well-known function of mTOR in cell size control (Zhang et al., 2000; Fingar et al., 2002). Meanwhile, the expression of *mTOR* shRNAs resulted in a decrease of the cytoophidium length in GFP-positive clones as compared to the normal cytoophidium formation observed in their neighboring cells (Fig. 6C‒G and I). Statistical analysis showed that the length of cytoophidia is less than 50% of the length of cytoophidia in their neighboring cells, suggesting that mTOR is required for cytoophidium assembly *in vivo*.

## 3 Discussion

A significant finding presented here is the connection between the mTOR pathway and CTPS cytoophidium assembly. mTOR has emerged as an important regulator of nucleotide metabolism (Ben-Sahra et al., 2013, 2016; Robitaille et al., 2013) and is implicated in multiple human cancer types (Saxton and Sabatini, 2017). Mutations in *mTOR* itself are observed in various cancer subtypes (Sato et al., 2010; Grabiner et al., 2014). mTOR also serves as a downstream effector for many frequently mutated prooncogenic pathways, such as Ras/Raf/MAPK pathway, resulting in the hyperactivation of mTOR pathway in numerous human cancers. However, single-agent therapies using mTORC1 inhibitors, including rapamycin and everolimus, only showed limited anti-cancer activity, mainly due to the inhibition of mTORC1 generally has cytostatic but not cytotoxic effects in cancer cells. Elevated CTP levels and increased CTPS enzyme activity have been reported in many types of cancer such as hepatomas, leukemia and colorectal cancer (Williams et al., 1978; Kizaki et al., 1980; Weber et al., 1980; Ellims et al., 1983; van den Berg et al., 1993). Knockdown of CTPS reduced tumorigenesis in a *Drosophila* tumor model (Willoughby et al., 2013), indicating that CTPS plays a functional role in tumor metabolism. In fact, CTPS has been an attractive anti-cancer target for decades. However, treatment with CTPS inhibitors such as acivicin and 6-Diazo-5-oxo-L-norleucine (DON) often provokes some unacceptable side effects, such as neurotoxicity, nausea and vomiting, which has hindered their further applications (Lynch et al., 1982; Rubin et al., 1983; Earhart et al., 1990; Falkson et al., 1990; Maroun et al., 1990). A recent study also reported that inactivation of CTPS caused imbalance of dNTP pools and increased mutagenesis in *Saccharomyces cerevisiae* (Schmidt et al., 2017). The assembly of CTPS into cytoophidium has been suggested as a way for modulating its enzymatic activity. Polymerization of CTPS inhibits its catalytic activity in *S. cerevisiae* and *Escherichia coli* (Barry et al., 2014; Noree et al., 2014). However, an *in vitro* study showed that filamentation of CTPS increases its enzymatic activity (Lynch et al., 2017).

We recently reported the increased abundance of CTPS cytoophidium in various human cancers including colon, prostate and live cancers (Chang et al., 2017). A larger nucleotide pool is required to support fast cancer cell growth. The potential advantage of the cytoophidium formation is to increase enzyme activity rapidly, provided that polymerization is faster than transcription. Inhibition of the rate-limiting enzyme in guanylate nucleotide synthesis, inosine monophosphate dehydrogenase (IMPDH), selectively kills mTORC1-activated cancer cells, implying that targeting nucleotide metabolism is promising for treating tumors with elevated mTOR signaling (Valvezan et al., 2017). Therefore, it will be interesting to determine whether inhibition of CTPS filamentation could suppress mTOR hyperactive cancer cell growth in future studies. If this is true, further identification of small molecules to disrupt CTPS polymerization may be a promising strategy for combating mTOR-driven cancers.

The mechanisms by which mTOR pathway controls CTPS filamentation is likely through direct and indirect manners. In this study, we understand that the regulation of CTPS filamentation seems not through reducing CTPS protein expression, which has been recognized as a critical factor for cytoophidium assembly (Ben-Sahra et al., 2013). We showed evidence supporting that the regulation of cytoophidium formation by mTOR pathway is being carried out mainly by S6K1 kinase. When *S6K1* was knocked down by siRNA, there is an approximate 50% reduction in the number of cells containing cytoophidia (Fig. 5A and B), and a further reduction in the percentage of cells stably expressing *S6K1* shRNA (Fig. 5D and E). Importantly, exogenous expression of a constitutively active S6K1 mutant (CA-S6K1) rescued *mTOR* knockdown-induced cytoophidium disassembly (Fig. 5G and H). Both transcriptional and post-transcriptional mechanisms especially phosphorylation can regulate CTPS enzymatic activity (Park et al., 1999, 2003; Choi et al., 2003; Han et al., 2005; Chang et al., 2007; Higgins et al., 2007). Indeed, filamentous CTPS can be recognized by a phospho-specific antibody against CTPS phosphorylated on serine 36 (Liu, 2010), whose mutation causes a decrease in CTPS catalytic activity (Park et al., 2003). These findings raised the possibility that phosphorylation could regulate CTPS activity by influencing cytoophidium assembly. Interestingly, a previous phosphoproteomics study identified several phosphorylation sites at the C-terminal of CTPS in the mTOR pathway-activated mouse embryonic fibroblasts (MEFs) (Robitaille et al., 2013). We recently reported that deletion of the conserved N-terminal of *Drosophila* CTPS, targets of multiple post-translational modifications including phosphorylation, is sufficient to interfere with cytoophidium assembly (Huang et al., 2017). Therefore, it will be interesting to determine if phosphorylation has a direct effect on CTPS filamentation in the future.

mTOR plays a central role in regulating cell size, cell cycle progression and cell proliferation in *Drosophila* and many other species (Zhang et al., 2000; Fingar et al., 2002; Lloyd, 2013). A previous study reported that reduction of CTPS could result in a decrease in nuclear size in *Drosophila* follicle cells (Wang et al., 2015). Our recent investigation in *Drosophila* also showed that CTPS is required for Myc-dependent cell size control (Aughey et al., 2016). It is worth to note that several nucleotide metabolizing enzymes are phosphorylated or upregulated in response to mTOR activation in mammalian cells leading to increased intracellular pools of pyrimidines and purines for DNA and RNA synthesis (Ben-Sahra et al., 2013, 2016; Robitaille et al., 2013). It is reasonable to believe that CTPS is involved in the regulation of nucleotide metabolism by mTOR, as CTP is essential for the biosynthesis of DAN and RNA. Although mounting evidence suggests that mTOR regulates nucleotide metabolism in cultured cells and tumor models (Ben-Sahra et al., 2013, 2016; Robitaille et al., 2013; Valvezan et al., 2017), the relevance of this relationship in normal animal development has not been well defined. In this study, we observed a connection between mTOR expression and the length of CTPS cytoophidium in *Drosophila* oogenesis.

Together, using the colorectal cancer cell line SW480 and *Drosophila* as model systems, we show that the mTOR pathway regulates CTPS cytoophidium assembly. We have found that pharmacological inhibition of the mTOR pathway or knockdown of mTOR protein expression significantly reduces cytoophidium formation without affecting CTPS protein expression. In addition, the mTOR pathway controls CTPS cytoophidium assembly mainly via the mTORC1/S6K1 signal axis. Collectively, our results show a connection between the mTOR pathway and CTPS cytoophidium assembly.

## 4 Materials and methods

### 4.1 Regents and antibodies

Antibodies for CTPS (15914-1-AP), Raptor (20984-1-AP), Rictor (27248-1-AP), S6K1 (14485-1-AP), ATF4 (10835-1-AP) and MTHFD2 (12270-1-AP) were purchased from ProteinTech (China). Antibodies for mTOR (#2983S) and Phospho-S6K1 (#9205) were purchased from Cell Signaling Technology (USA). Antibodies for c-Myc (ab32072) and β-Actin (ab6276) were purchased from Abcam (USA). Antibody for HA (sc-7392) and normal mouse IgG (sc-2025) were from Santa Cruz Biotechnology (USA). Antibody for α-tubulin (T5168) was purchased from Sigma-Aldrich (USA). Antibodies for *Drosophila* CTPS (134457 and 33304) were purchased from Santa Cruz. Antibody for Hu-li tao shao (Hts) (7H9 1B1) was purchased from Developmental Studies Hybridoma Bank (USA). Ramaycin (S1039) and everolimus (S1120) were from Selleck Chemicals (China).

### 4.2 Cell culture

293T, SW480 and NCM460 cells were cultured in Dulbecco's modified Eagle's medium (DMEM, SH30022.01, Hyclone, China), whereas LoVo, RKO, DLD-1 and HCT116 cells were cultured in Roswell Park Memorial Institute Medium 1640 (RPMI 1640, SH30809.01, Hyclone) supplemented with 10% fetal bovine serum (04-001, Biological Industries, Israel) and antibiotics (100 U/mL penicillin and 100 mg/mL streptomycin, and SV30010, Hyclone), in a humidified atmosphere containing 5% CO_2_ at 37 °C (normal culture conditions). Cell transfections were carried out by using Lipofectamine 2000 (11668019, Invitrogen, USA) or R0531 (Thermo Fisher Scientific, USA) according to the manufacturer's instructions.

### 4.3 *Drosophila* husbandry 

All stocks were maintained on standard *Drosophila* medium at 25 °C. *w*^*1118*^ (Bloomington stock centre) was used as a wild-type control in all our experiments. All RNAi stocks were from the TRiP collection (Bloomington Stock Center, USA).

### 4.4 Lentiviral shRNA cloning, production, and infection

Desalted oligonucleotides were cloned into pPLK/GFP + Puro purchased from the Public Protein/Plasmid Library (Nanjing, China) with the *Bam*HI/*Eco*RI sites at the 3′ end of the human *H1* promoter. The target sequences for *mTOR*, *Raptor* and *Rictor* are 5′-CCGCATTGTCTCTATCAAGTT-3′, 5′-CGAGTCCTCTTTCACTACAAT-3′ and 5′-CCGCAGTTACTGGTACATGAA-3′, respectively. The target sequences for *S6K1* are 5′-AGCACAGCAAATCCTCAGACA-3′ and 5′- CCCATGATCTCCAAACGGCCA -3′. Plasmids were propagated in and purified from top 10 bacterial cells and co-transfected together with psPAX2 and pMD2.G into HEK 293T cells. Virus-containing supernatants were collected at 48 h after transfection, and then filtered with 0.45 μm PES filters (Millipore, USA). Cells were infected with appropriate lentiviruses in the presence of 8 μg/mL polybrene (Millipore) for 48 h. The GFP-positive cells were purified by flow cytometry and then cultured in normal medium containing 0.5 μg/mL puromycin for 1 week. The resulting puromycin-resistant cells were used for further analysis.

### 4.5 siRNA and transfection

Small interfering RNA (siRNA) duplexes against *mTOR* (stQ0004935-1), *Raptor* (stQ0012651-1), *Rictor* (stQ0016785-1), *S6K1* (stQ0004595-1), *ATF4* (stQ0005631-1) and *MTHFD2* (stQ0002930-1) were purchased from Ribobio (Guangzhou, China). Three siRNA duplexes were used for one target gene to achieve greater knockdown efficiency and lower off-target effects. A 2 μL or 5 μL aliquot of 20 μM siRNA per well was transfected into cells seeded in 24-well or 6-well plates, respectively, with Lipofectamine 2000 (Invitrogen) according to the manufacturer's protocol.

### 4.6 Immunofluorescence

For mammalian, cells were cultured on glass slides and fixed with 4% paraformaldehyde in PBS for 10 min, and then permeabilized with 0.1% Triton X-100 for 10 min at room temperature. After washed with PBS, samples were blocked with 5 mg/mL bovine serum albumin in PBS for 1 h, followed by incubation with anti-CTPS antibodies overnight at 4 °C. After the primary antibody reaction, samples were washed and incubated with FITC-labeled secondary antibodies for 1 h. Finally, samples were washed and mounted with medium containing 4′,6-diamidino-2-phenylindole (DAPI), which was used to visualize nuclei. The images were taken under a confocal laser scanning microscope (Carl Zeiss, German).

For *Drosophila*, tissues were dissected into Grace's Insect Medium, and then fixed in 4% paraformaldehyde for 10 min. After that, tissues were washed with PBT (1× PBS + 0.5% horse serum + 0.3% Triton X-100), followed by overnight incubation with primary antibodies at room temperature. After primary antibody reaction, tissues were washed with PBT, and then incubated at room temperature overnight in secondary antibodies. Nuclei were labeled by Hoechst 33342. All samples were imaged using a Leica SP5II confocal microscope.

### 4.7 Western blotting

Cell lysates were prepared with NP-40 lysis buffer (150 mmol/L NaCl, 1.0% NP-40, 50 mmol/L Tris (pH 8.0)), and equal amounts of lysates were electrophoresed on a 10% SDS-PAGE gel. PVDF membranes (Roche) were used for protein transfer. The membranes were then blocked with 5% nonfat milk in TBST (150 mmol/L NaCl, 50 mmol/L Tris-HCl (pH 7.4), and 0.1% Tween 20) for 1 h, followed by incubation with appropriate primary antibodies at 4 °C overnight. After primary antibody reaction, the membranes were washed with TBST three times and then incubated with HRP-labeled secondary antibody at room temperature for 1 h. After washed again with TBST for three times, the signals of secondary antibodies were detected by an enhanced chemiluminescence system.

### 4.8 RNA extraction and quantitative real-time PCR

Total RNAs were extracted by Trizol (Invitrogen). The first-strand cDNA synthesis was conducted with RevertAid First-Strand cDNA synthesis kits (Fermentas, USA). qRT-PCR reactions were performed using SYBR Green dye and the Applied Biosystems 7500 Fast Real-Time PCR System. Primers used for *c-Myc* (forward primer, 5′-GGCTCCTGGCAAAAGGTCA-3′; reverse primer, 5′-CTGCGTAGTTGTGCTGATGT-3′), *Cbl* (forward primer, 5′-TGGTGCGGTTGTGTCAGAAC-3′; reverse primer, 5′-GGTAGGTATCTGGTAGCAGGTC-3′) and *β-actin* (forward primer, 5′-CATGTACGTTGCTATCCAGGC-3’; reverse primer, 5′- CTCCTTAATGTCACGCACGAT-3′). The resulting values were normalized to *β-actin* expression.

### 4.9 Co-immunoprecipitation assay

For Co-IP assay, SW480 cells stably expressing HA-CA-S6K1 were cultured in 10 cm dishes for 48 h, and then cell lysates were prepared with Co-IP lysis buffer (Hepes-NaOH 50 mmol/L (pH7.5), NaCl 100 mM, EDTA 2.5 mM, NP-40 0.5%, DTT 1 mmol/L and proteasome inhibitors). Cell lysates were incubated with the appropriate antibody for 1h, and subsequently incubated with protein A-Sepharose beads overnight at 4 °C. The protein-antibody complexes recovered on beads were subjected to Western blotting using appropriate antibodies after separation by SDS-PAGE.

### 4.10 Statistical analysis

Two-tailed unpaired Student's *t*-test was used for comparisons between two groups and ordinary one-way ANOVA with Tukey's multiple comparison post-test was used to compare variables among three or more groups. The quantification of the percentage of cells containing cytoophidia was from at least three independent experiments, and more than 200 cells were counted for each quantification. *P* ≤ 0.05 was considered statistically significant. All analyses in human cells were performed using GraphPad Prism version 6.00 (GraphPad Software, San Diego, CA, USA, www.graphpad.com). For *Drosophila* data, image processing and analysis was conducted using Leica Application Suite Advanced Fluorescence Lite and ImageJ. Each group over 60 *Drosophila* follicle cells were quantified. Nuclear sizes or the length of cytoophidia are expressed as a ratio of the average nuclear size or cytoophidium length in GFP marked clones to neighbouring cells (GFP negative).
